# Torula yeast in the diet of Atlantic salmon *Salmo salar* and the impact on growth performance and gut microbiome

**DOI:** 10.1038/s41598-021-04413-2

**Published:** 2022-01-12

**Authors:** Alexandra Leeper, Ricardo Ekmay, Stephen Knobloch, Sigurlaug Skírnisdóttir, Madhushri Varunjikar, Marianne Dubois, Birgir Örn Smárason, Jón Árnason, Wolfgang Koppe, David Benhaïm

**Affiliations:** 1 Department of Animal and Aquaculture Sciences, Faculty of Biosciences, Norwegian University of Life Sciences, P.O. Box, 1420 Ås, Norway; 2 Department of Research and Innovation, Matís Ltd, 12, Vínlandsleid, 113 Reykjavik, Iceland; 3 Department of Aquaculture and Fish Biology, Hólar University, Haeyri 1, 551 Saudárkrókur, Iceland; 4 Arbiom Inc., Durham, NC 27703 USA

**Keywords:** Applied microbiology, Ichthyology

## Abstract

Atlantic salmon aquaculture is expanding, and with it, the need to find suitable replacements for conventional protein sources used in formulated feeds. Torula yeast (*Cyberlindnera jadinii*), has been identified as a promising alternative protein for feed and can be sustainably cultivated on lignocellulosic biomasses. The present study investigated the impact of torula yeast on the growth performance and gut microbiome of freshwater Atlantic salmon. A marine protein base diet and a mixed marine and plant protein base diet were tested, where conventional proteins were replaced with increasing inclusion levels of torula yeast, (0%, 10%, 20%). This study demonstrated that 20% torula yeast can replace fish meal without alteration to growth performance while leading to potential benefits for the gut microbiome by increasing the presence of bacteria positively associated with the host. However, when torula yeast replaced plant meal in a mixed protein diet, results suggested that 10% inclusion of yeast produced the best growth performance results but at the 20% inclusion level of yeast, potentially negative changes were observed in the gut microbial community, such as a decrease in lactic acid bacteria. This study supports the continued investigation of torula yeast for Atlantic salmon as a partial replacement for conventional proteins.

## Introduction

Aquaculture production is predicted to be increasingly important for global food security^1^. In Europe, Atlantic salmon (*Salmo salar*) aquaculture continues to grow in both market share and production intensity^2^. Modern formulated feeds have developed from relying heavily on fish meal (FM), primarily sourced from wild capture fisheries but have evolved to incorporate large quantities of plant protein sources such as soybean, rapeseed and corn meals^3^. The scale of finfish production now necessitates the integration of alternative sources of dietary protein. The next generation of feed ingredients must meet the nutritional requirements of this carnivorous species, while alleviating market competition for terrestrial agriculture products and negating the adverse impact on growth and gut health attributed to anti-nutritional factors (ANFs) present in many plant ingredients^4^.

Torula yeast (*Cyberlindnera jadinii,* anamorph name *Candida utilis*) has long been considered for animal feed, historically as a functional feed additive, and more recently as a protein source^5,6^. Existing research has investigated the potential to include torula yeast in diets of poultry^7^, pigs^8–10^, and a wide range of aquaculture species including, Pacific white shrimp (*Litopenaeus vannamei*)^11^, Nile tilapia (*Oreochromis niloticus*)^12^ and Atlantic salmon^13,14^. As a protein source, torula yeast has a high potential value since it can be cultivated independent of location and climate and does not add to pressure on existing agricultural systems^5^. It can even be cultivated on side streams such as lignocellulosic biomasses, which are often low-value non-food wastage from other industries like forestry. This produces ingredients with high protein content and value to aquaculture and could support circular bioeconomic growth^15–17^. Research attention has identified yeast as a promising candidate to replace FM and plant protein sources such as soybean meal (SBM) in feed as demand for alternative protein sources rises^18,19^.

The case for inclusion in aquaculture feeds for Atlantic salmon (*S. salar*) is compelling. Torula yeast has a suitable protein content and amino acid profile for fish feed^5,15,20^. In freshwater (FW) stage Atlantic salmon, torula yeast has successfully replaced up to 40% of the crude protein from FM compared with a control of 58% FM without negative impact on growth performance^18^. Torula yeast has displayed beneficial functional properties such as a reduction in inflammation of the distal intestine during smoltification, a crucial developmental period for Atlantic salmon^14^. Torula yeast has also been associated with immune-modulating benefits which could greatly enhance their value to the aquaculture industry^21^. However, a significant research gap exists in optimising and standardising the dietary inclusion levels which must be addressed to facilitate commercial adoption of this alternative protein^22^.

The gut microbiome of cultured fish species plays a key role in many aspects of health, welfare, immune development^23^, disease resistance^24^, growth^25^, digestion and nutrient uptake^26,27^. Multiple factors influence the establishment and final composition of the gut microbiome in farmed Atlantic salmon, one key factor is the diet of the host^25,28,29^. Additional important factors include, the life stage of the fish and the surrounding environment^30,31^. Consequently, it is important to consider changes to gut microbiome composition and their potential consequences when investigating the suitability of new and alternative feed ingredients for aquaculture^32^. The gut microbiome during early life stages of fish, and the impact of changing diet is of particular interest since it can influence the development of adult gut microbiome and therefore overall development^33,34^. During early life stages, the gut microbiome is highly malleable to dietary influence^35,36^. The presence of certain bacteria have functional immune and development benefits to the fish, for example, lactic acid bacteria (LAB) and bacilli^37^, whereas dysbiosis or an unbalanced microbial community is associated with undesirable conditions and consequently, poor health^38,39^.

When plant protein replaces or partially replaces marine protein in salmonid diets, the gut microbiome is significantly altered. Pea protein replacing 10% FM increased *Streptococcus*, *Leuconostoc*, and *Weissella,* which are LABs^29^*,* and mixed plant meals replacing 97% mixed animal and marine meals increased Lactobacillales*, *Bacillales and Pseudomonadales, compared with Bacteroidales, Clostridiales, Vibrionales, Fusobacteriales and Alteromonadales^35^ in juvenile Rainbow Trout (*Oncorhynchus mykiss*). Atlantic salmon post-smolts fed 30% inclusion levels of soybean meal and soy protein concentrate had higher relative abundances of LABs than those fed a fish meal diet^32^*.* When the yeasts, *Saccharomyces cerevisiae* and *Wickerhamomyces anomalus* replaced 40% and 60% of fish meal in Rainbow trout (*O. mykiss*) there was a significant alteration of the gut microbiome, whereas, 20% *S. cerevisiae* did not alter the community^40^. In pig diets, torula yeast was associated with selection for *Lactobacillus *spp. in the gut microbiome^41^. There exists a gap in the literature clarifying how torula yeast affects fish gut microbiome despite the growing interest in this protein for dietary inclusion and this is important to characterise the impact of this alternative ingredient for the aquafeed industry^5^.

The aim of the present study was to evaluate the effect of replacing conventional proteins with increasing inclusion of torula yeast (*C. jadinii*) on both growth performance and the microbiota present in the gastro-intestinal (GI) tract of juvenile Atlantic salmon. To optimise this characterisation and provide a comprehensive investigation two basal diets were considered. The first, was a marine protein-based diet where fish meal (FM) was replaced with increasing levels of torula yeast to provide a simplified replacement. The second, a marine protein and plant protein-based combination where a mixture of plant meals (MIX) was replaced with increasing levels of torula yeast to provide a commercially relevant dietary replacement. These two basal diets were formulated separately thus, will be investigated here separately.

## Methods

### Experimental animals and study design

Atlantic salmon (*S. salar*) hatching on 31 October 2018 were reared at 5.5 °C by Stofnfiskur Ltd. (Iceland). Eyed eggs were transferred to Laxar ehf. (Iceland) where they were raised to first feeding using standard commercial techniques and commercial start-feed diet BioMar Inicio-plus (United Kingdom) of 0.5mm pellet size. Fry were transferred to Matís Aquaculture Research Station (MARS) on 23 January 2019, where they were acclimated for one week to the study facilities. All fish within the experiment were individually weighed following a 12-h fasting period under anaesthetic (2-phenoxyethanol of 300 ppm). At the beginning of the feeding trial fish weight was1.14 ± 0.1 g. Fish were split into eighteen 20 L-white circular PVC tanks, in triplicate for each feed treatment. Each tank contained 20 individual fish. Fish were kept in freshwater at 9.5 ± 0.5 °C under continuous light (20 ± 4 lux), oxygen levels were maintained above 80% saturation. Fish were fed with the experimental feed treatments for 35 days continuously. The experiment was performed following European and Icelandic guidelines and within the permits and licences of the MARS facility as described in the *Ethical declarations* of this manuscript. The experimental protocols were approved by Matís ohf, Iceland and under the licence FE-1134 (Rekstrarleyfi) from MAST and UST201707 (Starfsleyfi) from the Icelandic Environment Agency. The experimental design also complied with the ARRIVE guidelines.

### Production of torula yeast

Dried, inactive torula yeast (*C. jadinii*) was cultivated by RISE Processum (Borås, Sweden) on wood hydrolysates provided by Arbiom Inc. (Durham, NC, USA). Briefly, wood hydrolysates were generated from hardwood chips (mixed species) locally sourced from Virginia, USA. Subsequently, the hydrolysates were fermented in a 50-L reactor under continuous operation followed by washing, thermal inactivation, and drying to produce the final product.

### Experimental feed treatments and feeding

The two basal diets were formulated for this investigation. In the first basal diet group there were three treatments with a marine protein base, representing a simplified replacement where fish meal (FM) was increasingly replaced with torula yeast with inclusion levels 0% (FM00), 10% (FM10), and 20% (FM20) (Table 1). In the second basal diet group there were three treatments with a marine protein and plant protein combination base, representing a commercially relevant replacement, where a plant protein mixture (MIX) was increasingly replaced with torula yeast with inclusion levels 0% (MIX00), 10% (MIX10), and 20% (MIX20) (Table 2). The chemical composition of all protein sources used in feeds for this experiment from both FM and MIX basal diets are presented (Table 3). The feed treatments were produced by cold pelleting at Matís ohf. (Iceland), with a pasta machine (ADE, Germany). All dry ingredients were milled to bring all materials to equal particle size (IPHARMACHINE, Germany). Dry ingredients were then homogenised in a standard food mixer (KitchenAid, USA) and re-milledto improve the homogeneity of the feed. The dry mix was returned to the food mixer and fish oil was added while simultaneously mixing, a small volume of water was added to produce the ideal consistency for the next stage (200ml). The mix was then processed in the pasta machine to produce 0.5mm pellets. These pellets were dried in a commercial food dryer (Kreuzmayr, Austria) to <10% moisture content. Resulting feed treatments were analysed for chemical composition (Table 1 FM, Table 2 MIX). During the 35-day feeding trial, fish were fed 5 times per day by electric belt-feeder between the hours of 08:00 to 20:00. All fish were fed identical volumes, with 15% excess based on feed requirements at this developmental stage.

**Table 1:** Feed formulation and chemical composition for marine protein (FM) feed treatments.

	FM0%	FM10%	FM20%
**Formulation (g kg**^**−1**^**)**			
Fish meal^a^	676.8	610.0	542.9
Pre-gelatinised wheat^b^	209.7	172.9	136.2
Vitamin-mineral premix^c^	10.0	10.0	10.0
Fish oil^a^	103.5	107.1	110.8
Torula yeast (*Cyberlindnera jadinii*)	0.0	100.0	200.0
**Analysed content (g kg**^**−1**^**)**			
Dry matter	935	932	936
Crude protein	506	502	507
Crude lipid	162	165	170
Ash	103	100	97
**Essential amino acids (g kg**^**−1**^**)**			
Arginine	22.2	27.6	27.9
Histidine	9.3	11.5	11.6
Isoleucine	15.7	19.9	20.7
Leucine	29.3	36.4	37.2
Lysine	29.7	37.4	37.2
Methionine	12.1	12.8	11.3
Phenylalanine	15.5	19.4	19.6
Threonine	17.4	21.4	22.2
Valine	19.5	24.4	25.4
Tryptophan	5.5	5.5	5.2
**Non-essential amino acids (g kg**^**−1**^**)**			
Alanine	23.6	29.2	30.6
Aspartic acid	36.1	43.8	44.9
Glycine	24.9	30.3	30.6
Glutamic acid	56.0	69.8	70.5
Cystein + cysteine	4.5	5.3	5.2
Tyrosine	12.6	15.7	15.8
Proline	17.0	21.7	19.4
Serine	17.2	21.3	22.4

^a^Laxá hf. Krossanes, Iceland.

^b^Emmelev A/S, Denmark.

^c^Laxa salmon premix 2006 1%, Trouw Nutrition, The Netherlands.

**Table 2:** Feed formulation and chemical composition for marine and plant protein combined (MIX) feed treatments.

	MIX0%	MIX10%	MIX20%
**Formulation (g kg**^**−1**^**)**			
Fish meal^a^	425.0	425.0	425.0
Pre-gelatinised wheat^b^	191.1	159.4	127.7
Corn gluten meal^a^	72.5	53.2	33.9
Vitamin-mineral premix^c^	10.0	10.0	10.0
Fish oil^a^	116.7	116.8	117.0
Torula yeast (*Cyberlindnera jadinii*)	0.0	100.0	200.0
Lysine-HCl	8.6	6.0	3.4
DL-methionine	0.4	0.7	1.0
Soy protein concentrate^a^	75.7	55.5	35.4
Wheat gluten meal^a^	100.0	73.3	46.7
**Analysed content (g kg**^**−1**^**)**			
Dry matter	942	918	935
Crude protein	515	495	506
Crude lipid	154	162	161
Ash	79	80	86
**Essential amino acids (g k**^**−1**^**)**			
Arginine	25.7	23.5	31.7
Histidine	11.5	10.1	11.6
Isoleucine	20.1	17.6	20.8
Leucine	41.1	35.3	39.0
Lysine	34.4	29.4	32.7
Methionine	11.9	11.1	11.4
Phenylalanine	20.8	18.7	21.4
Threonine	19.6	17.7	20.6
Valine	23.6	21.2	25.3
Tryptophan	5.2	5.2	5.6
**Non-essential amino acids (g k**^**−1**^**)**			
Alanine	28.0	25.0	29.9
Aspartic acid	40.4	36.0	43.4
Glycine	26.4	24.1	28.2
Glutamic acid	92.9	77.7	81.4
Cystein + cysteine	6.2	6.1	6.1
Tyrosine	16.6	14.1	17.4
Proline	29.5	24.6	23.6
Serine	22.4	19.8	23.4

^a^Laxá hf. Krossanes, Iceland.

^b^Emmelev A/S, Denmark.

^c^Laxa salmon premix 2006 1%, Trouw Nutrition, The Netherlands.

**Table 3:** Chemical composition of the protein sources used in feed treatments of this study.

Composition (g kg^−1^)	Raw protein materials				
	Torula yeast (*Cyberlindnera jadinii*)	Fish meal	Soy protein concentrate	Corn gluten meal	Wheat gluten meal
Dry matter	938.0	909.0	925.0	910.0	922.0
Crude protein	514.0	659.0	633.0	582.0	742.0
Crude lipid	25.1	107.0	2.0	10.0	16.0
Ash	88.4	139.0	90.0	23.0	11.0
**Essential amino acids (g kg**^**−1**^**)**					
Arginine	30.8	42.3	42.9	18.6	25.0
Histidine	8.9	18.2	15.5	12.2	14.0
Isoleucine	20.2	30.2	27.5	23.2	24.5
Leucine	32.0	57.1	47.4	96.1	48.3
Lysine	34.2	58.8	37.0	9.1	11.4
Methionine	5.3	19.6	9.0	14.3	11.8
Phenylalanine	18.6	28.9	32.1	37.2	35.7
Threonine	22.9	33.3	25.1	20.3	17.8
Valine	26.0	37.8	28.5	28.0	27.4
Tryptophan	5.8	7.6	8.6	3.1	7.5
**Non-essential amino acids (g kg**^**−1**^**)**					
Alanine	28.4	47.2	26.3	52.7	18.1
Aspartic acid	39.8	69.2	71.3	36.2	21.9
Glycine	21.3	50.3	25.6	17.4	23.3
Glutamic acid	66.1	105.0	114.0	130.0	260.0
Cysteine (+ cysteine)	3.3	5.8	8.6	11.4	15.5
Tyrosine	16.1	24.6	22.3	30.6	22.9
Proline	15.0	32.1	30.9	55.7	86.9
Serine	21.0	32.6	32.4	33.5	34.7

### Growth performance

After 35 days of continuous feeding all individual fish from all FM and MIX tanks were weighed (wet weight (g)) and measured (total length (cm) following a 12-h fast. From this data the Fulton´s condition factor (K) and specific growth rate (%) (SGR) over the study period could be calculated: K = (Weight/Total Length^3^) × 100 and SGR = ((Ln(Final Weight) − Ln(Initial Weight)) × 100)/*t*, where $t$ is the number of days over which the trial was run. Mortality was monitored daily throughout the feeding trial.

### Gut sampling

After the growth performance assessment, all fish were left for one week to recover from handling and fed with the respective FM and MIX feed treatments. At the end of this week fish were fasted for 12 h and then randomly sampled, three (3) fish from each tank, nine (9) fish per feed treatment. Sampled fish were euthanised with a lethal dose of anaesthetic (phenoxyethanol 600 ppm) and the fish was rinsed in 90% ethanol followed by sterile distilled water. The GI tract from the top of the mid-gut, just below the pyloric caeca down to the end of the distal gut, was directly removed under sterile conditions, with any content present included. Samples were then stored at −80 °C prior to downstream processing.

### DNA extraction, PCR amplification and sequencing

Gut samples were individually homogenised, manually using a sterile petri dish with a sterile scalpel to break up the gut sample. Samples were transferred to a sterile 2 ml Eppendorf tube with 300 µl of sterile 1mm diameter sterile silica beads (BioSpec Products, United States). 800µl of CD1 solution from the QIAamp PowerFecal Pro DNA kit (QIAGEN, Germany) was added to the Eppendorf tube. Samples were vortexed for 5 s and shaken at maximum speed (30Hz) in a laboratory mixer mill (Retsch MM400) for 1 min. The supernatant (~ 800 µl) was transferred to the PowerBead Pro Tube from the QIAGEN QIAamp PowerFecal Pro DNA kit. The protocol for this DNA extraction kit was then followed and finally DNA was eluted with 80 µl of C6 solution. A negative control with no material was also run to ensure no contamination occurred during the DNA extraction protocol. DNA concentration was measured using 2 µl of sample in the Invitrogen Qubit dsDNA BR Assay kit (Invitrogen, Carlsbad, CA, USA) which measure samples with DNA concentration from 2 to 1000 ng. DNA were diluted to 4 ng/µl in a 50 µl aliquot. Samples were then subjected to PCR of a region covering V3–V4 regions of the 16S rRNA gene with a universal bacterial primer pair S-D-Bact-0341-b-S-17 (5′-CCTACGGGNGGCWGCAG-3′)/S-D-Bact-0785-a-A-21(5′-GACT-ACHVGGGTATCTAATCC-3′)^42^. The PCR master mix included the diluted DNA, nuclease-free water, Q5 High-Fidelity DNA polymerase (New England Biolabs, Ipswich, USA), Q5 GC Enhancer, 0.5 µM of each primer containing Illumina overhang adapters, and 1× Q5 Reaction buffer, 200 µM dNTPs (New England Biolabs, Ipswich, USA). Included in the PCR were both positive and negative samples to monitor for successful amplification and absence of contamination of the target region only. The thermocycling protocol had an initial denaturation step (98 °C for 30 s), then 35 cycles of, denaturation (98 °C for 10 s), annealing (52 °C for 30 s), and extension (72 °C for 30 s), with a final extension (72 °C for 2 min). Libraries were multiplexed with Nextera XT v2 barcodes (Illumina, USA), normalised using *Sequel-Prep* Normalisation Plates (ThermoFisher Scientific, USA) then sequenced on a MiSeq desktop sequencer (Illumina, USA) using v3 chemistry and 2 × 300 cycles.

### Statistical methods

#### Growth performance

Statistical analyses were performed in R version 3.6.1 (2019-07-05). All tests were two-tailed with a significance level set to α = 0.05. FM and MIX diets were considered separately since they were formulated independently but identical statistical designs were used.

For growth performance, two dependent variables were statistically assessed, Condition Factor (K) and SGR (%). A Generalised Linear Mixed Model (GLMM) model was assessed first with the package *lme4*^43^. Feed treatment was considered a fixed factor and tank replicate was considered a random nested factor of feed treatment. For FM and MIX diets the tank replicate did not have a significant effect. Therefore, a Generalised Linear Model (GLM) with the package *nlme*^44^ was selected where for FM and MIX diets respectively feed treatment was a fixed factor and tank effect was not significant. Tukey post-hoc testing was applied to results with significant output.

#### Gut microbiome

Demultiplexed FASTQ files from Illumina were processed to produce amplicon sequence variants (ASVs) using the dada2 package version 1.16.0^45^ in Rstudio version 4.0.2^46^. The function *filterAndTrim* set variables as, *truncLen*=c(280,250), *trimLeft=* 21*, maxN*=0, *maxEE*=c(2,2), *truncQ=*2 and the *learnError* function was performed on a subset of 105888913 reads. The SILVA database version 138 was used to *assignTaxonomy* to the ASVs^47^. The microbial community was analysed using R packages phyloseq^48^, microbiome^49^ and vegan^50^, and visualised with ggplot2^51^. The number of reads output from the dada2 pipeline were 15449.44 ± 3575.3 for fish meal-based diets and 13317.48 ± 3341.2 for mix meal-based diets. PCR and DNA negative control samples were included in the sequencing and dada2 pipeline to check for potential contamination of samples, there was no detectable contamination in these control samples. For comparison the read depth was normalised across samples with the function *rarefy_even_depth* to the sample with the lowest read depth. ASVs from the Kingdom Eukaryota, Order Chloroplast and Family Mitochondria were removed from downstream analysis as they are often remnants from 16S fragments in Eukaryotes and do not belong to the bacteria. Raw 16S rRNA gene amplicon reads are deposited in the Sequence Read Archive under BioProject PRJNA732903.

The microbiome community for FM and MIX basal diets were quantitatively analysed using alpha and beta diversity measures. The selected alpha diversity measures where the observed richness of ASVs, Shannon diversity, Chao1 diversity and Pielou’s Evenness. A GLMM was used to assess if there was a significant difference in these alpha diversity measures between the different feed treatments from the FM and MIX diets, respectively. In this model feed treatment was a fixed factor and tank was a nested random factor of feed treatment. The random nested factor of tank was tested by a likelihood ratio test (LRT)^52^. Post-hoc testing was carried out using Tukey test. The microbiome community assemblage for each of the feed treatment types were transformed using a *Bray-Curtis* dissimilarity matrix and non-metric multidimensional scaling was applied. An analysis of similarity (ANOSIM) test was applied to assess for significant difference between and within fish fed different feed treatments in the FM and MIX feeds respectively. To further investigate the microbiome community assemblage the relative abundance as a proportion was visualised at the phylum level in stacked bar plots for direct comparison. The genus level was then visualised, with all genera present at less than 1% abundance amalgamated into a category called “Other” and the genera present at greater than 1% abundance were visually displayed using boxplots for each feed treatment in the FM and MIX treatments respectively.

### Ethics declarations and approval for animal experiments

The trial was carried out under the licence FE-1134 (Rekstrarleyfi) from MAST and UST201707 (Starfsleyfi) from the Icelandic Environment Agency. The authors complied with the ARRIVE guidelines.

## Results

### Growth performance

**Figure 1:**
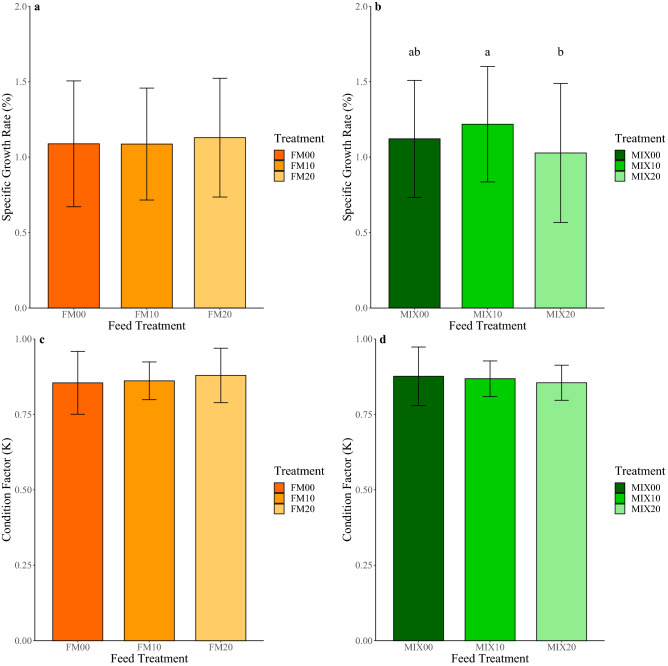
Bar plots of the specific growth rate (%) for (**a**) FM feed treatments and (**b**) MIX feed treatments, and bar plots of the condition factor (K) for (**c**) FM feed treatments and (**d**) MIX feed treatments. Error bars represent the standard deviation of the data and different lowercase letters indicate significantly different means (*P* < 0.05).

For FM diets, fish SGR (%) was not significantly different between the three inclusion levels of torula yeast with very similar average values and standard deviations (FM00: 1.09 ± 0.42, FM10: 1.09 ± 0.37, FM20: 1.13 ± 0.39) (Fig. 1a). For MIX diets, MIX10 had the highest SGR%. There was a significant difference in SGR % between MIX10 treatment and the highest inclusion MIX20. But there was no significant difference between the control MIX00 and either MIX10 or MIX20 (MIX00: 1.12 ± 0.39, MIX10: 1.22 ± 0.38, MIX20: 1.03 ± 0.46) (Fig. 1b).

For FM diets, the Condition Factor (K) was not significantly different between the three inclusion levels (FM00: 0.85 ± 0.10, FM10: 0.86 ± 0.06, FM20: 0.88 ± 0.09) (Fig. 1c). For MIX diets, the Condition Factor (K) was not significantly different between the three inclusion levels (MIX00: 0.88 ± 0.10, MIX10: 0.87 ± 0.06, MIX20: 0.85 ± 0.06) (Fig. 1d).

No mortality was observed in any feed treatment during the trial period.

### Gut microbiome

**Figure 2:**
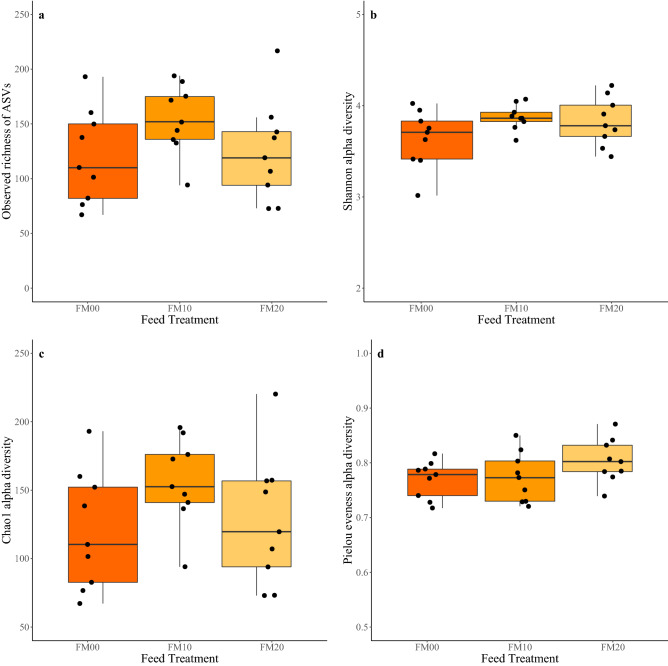
Box plots of alpha diversity measures for the FM feed treatments (**a)** the observed richness of ASVs, (**b)** Shannon diversity, (**c)** Chao1 diversity, (**d)** Pielou’s evenness.

**Figure 3:**
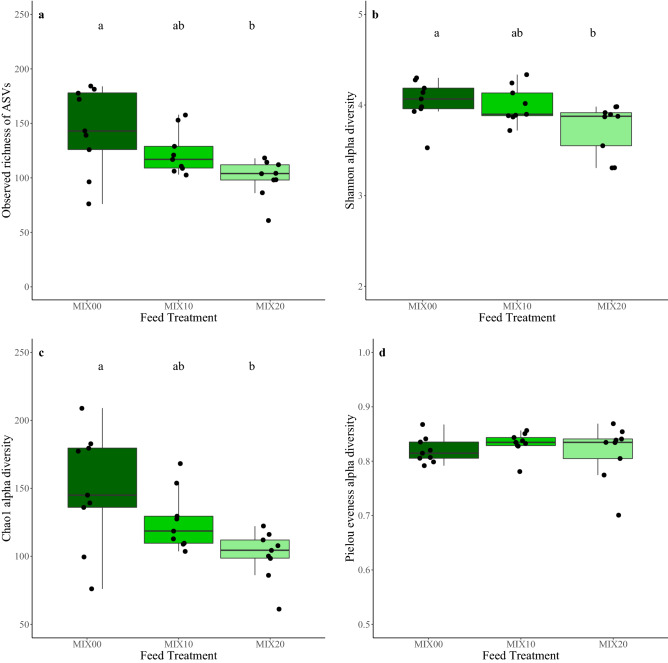
Box plots of alpha diversity measures for the MIX feed treatments (**a)** the observed richness of ASVs, (**b)** Shannon diversity, (**c)** Chao1 diversity, (**d)** Pielou’s evenness. Different lowercase letters indicate significantly different means (P < 0.05).

For FM diets, no significant difference was detected in any of the alpha diversity measures tested across the three inclusion levels of torula yeast. While not significant, FM10 fish did have a higher average value for Observed richness of ASVs (Fig. 2a), Shannon diversity (Fig. 2b), and Chao1 diversity (Fig. 2c) than other inclusion levels, but FM20 fish had the greatest community eveness (Fig. 2d). For MIX diets, three of the alpha diversity measures were significantly different. For the Observed richness of ASVs (Fig. 3a), Shannon diversity (Fig. 3b) and the Chao1 diversity (Fig. 3c), there was a significantly lower value for MIX20 than in the control, MIX00. There was no significance in the Pielou´s evenness between the three inclusion levels (Fig. 3d).

**Figure 4:**
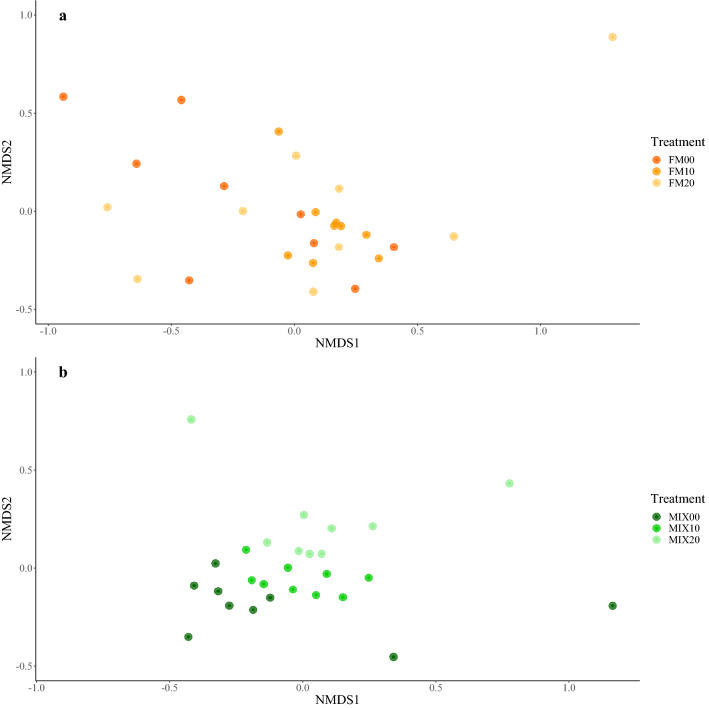
Non-metric multidimension scaling (NMDS) of fish from (**a)** FM feed treatments, (**b)** MIX feed treatments. Each point represents a single fish and colour indicates the feed treatment inclusion level.

**Figure 5:**
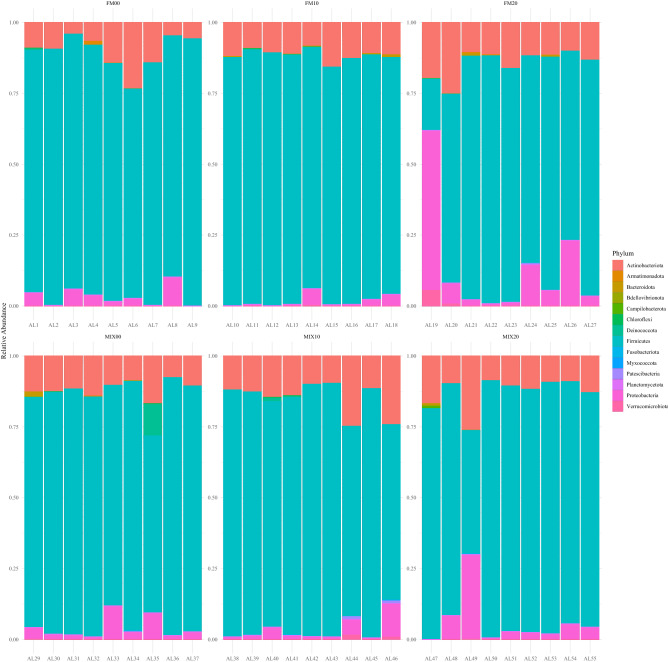
Stacked bar plot of gut bacterial composition using relative abundance of the most common phyla for fish from FM diets and fish from MIX diets. X axis shows sample names.

The microbial community composition for FM diets, were significantly different between all inclusion level, with greater difference between inclusion level than within it, ANOSIM P = 0.001 R = 0.2234 (Fig. 4a). For MIX diets, the microbiome community composition was significantly different between inclusion levels and there was a great difference between inclusion level than within it, ANOSIM P = 0.003 R = 0.1097 (Fig. 4b). At the phylum taxonomic level for the FM diets, the gut microbiome was dominated by Firmicutes for all inclusions of torula yeast (FM00 = 75.4% ± 6.3, FM10 = 85.54% ± 1.8, FM20 = 67.43 ± 20.6). The second and third most dominate phyla for all inclusions were Actinobacteria (FM00 = 9.28 ± 6.6, FM10 = 11.14 ± 2.1, FM20 = 13.60 ± 5.4) and Proteobacteria (FM00 = 3.07 ± 2.6, FM10 = 2.44 ± 2.2, FM20 = 13.11 ± 17.5) respectively. All other phylum occurring were present at 1% or lower relative abundance (Fig. 5). At the phylum taxonomic level for the MIX diets, the gut microbiome was dominated by Firmicutes for all inclusions of torula yeast (MIX00 = 75.73 ± 13.2, MIX10 = 77.58 ± 10.2, MIX20 = 73.78 ± 13.5). The second and third most dominate phyla for all inclusions were Actinobacteria (MIX00 = 10.82 ± 1.9, MIX10 = 14.08 ± 5.5, MIX20 = 11.9 ± 5.8) and Proteobacteria (MIX00 = 5.62 ± 2.5, MIX10 = 4.27 ± 3.4, MIX20 = 6.36 ± 9.0) respectively (Fig. 5).

**Figure 6:**
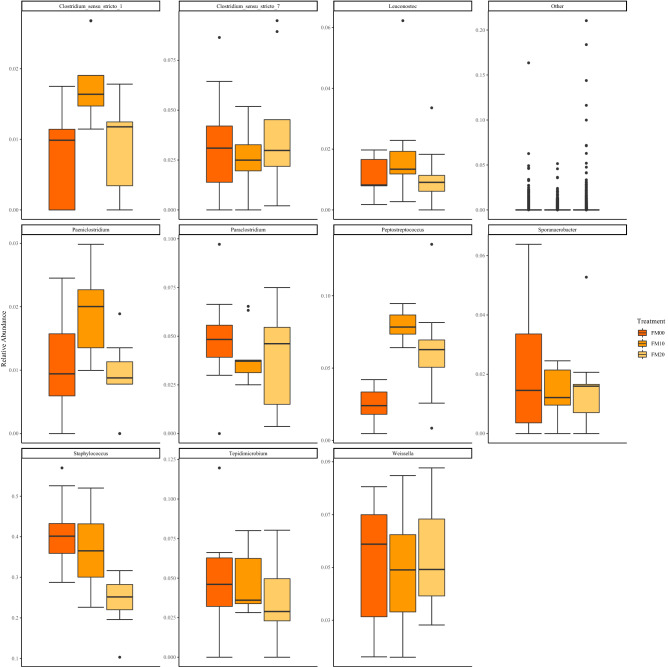
Boxplots of gut bacterial composition using relative abundance of the most common genera for FM diets.

**Figure 7:**
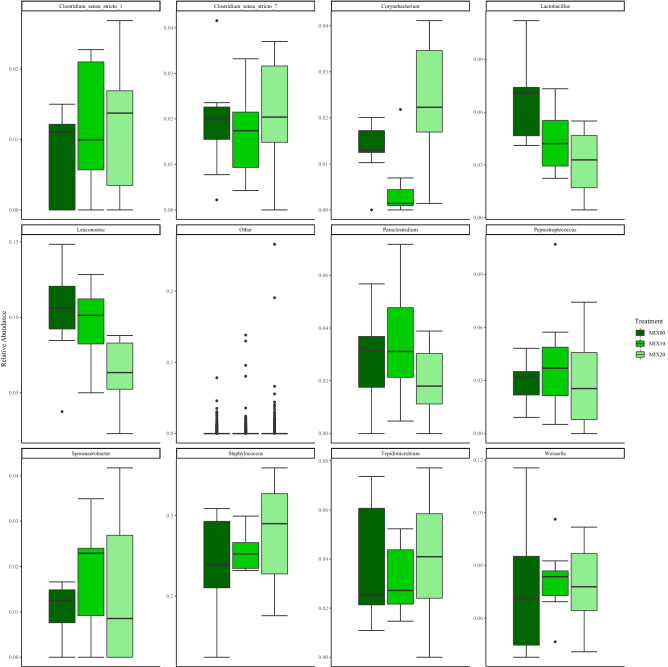
Boxplots of gut bacterial composition using relative abundance of the most common genera for MIX diets.

At the genus taxonomic level for the FM diets the genus *Staphylococcus* had the highest relative abundance, and abundance was lowest in FM20 compared with FM10 and FM00. For *Clostridium_sensu_stricto1*, *Leuconostoc*, *Preptostreptococcus* and *Paeniclostridium,* there is a pattern of greater average relative abundance in the FM10 inclusion level than both FM00 and FM20. The opposite trend was observed for *Sporanaerobacter*, *Paraclostridium* and *Clostridium_sensu_stricto7* which average relative abundance was slightly lower in FM10 compared with FM00 and FM20*.* There was decreasing presence of *Tepidmicrobium* with increasing inclusion of torula yeast. There was a similar average relative abundance of *Weisella* for inclusion levels but with a slight increasing trend with increasing torula yeast inclusion (Fig. 6). At the genus taxonomic level for the MIX diets the genus *Staphylococcus* had the highest relative abundance, and the average relative abundance increased with increasing inclusion of torula yeast, as did the average abundance of *Weissella*. Conversely, there was a trend of decreasing presence of *Lactobacillus* with increasing inclusion. *Corynebacterium* was highest in MIX10, and *Sporanaerobacter* was lowest in the same inclusion. *Tepidmicrobium* and *Clostridium_sensu_stricto_1* were highest in MIX20. Whereas, for *Peptostreptococcus*, *Paraclostridium* and *Leuconostoc* they were lowest in MIX20. There were similar levels of *Clostridium_sensu_stricto_7* at all inclusion levels (Fig. 7).

## Discussion

The existing literature has established the potential of torula yeast as a valuable protein source for the aquafeed industry^15,18^. The present study addressed essential knowledge gaps regarding the effect of replacing conventional proteins with increasing inclusions of torula yeast on growth performance and gut microbiome of FW Atlantic salmon (*Salmo salar*). Two separate base diets were investigated. A marine protein base diet where fish meal (FM) was replaced with increasing levels of torula yeast to provide a simplified replacement, and a marine protein and plant protein combination where mixed plant meals (MIX) were replaced with increasing levels of torula yeast to provide a commercial relevant dietary replacement.

Growth performance for fish fed the FM diets was comparable between all inclusion levels for both the SGR (%) and Condition Factor (K) up to 20% inclusion and fell within a normal range for FW Atlantic salmon^53^. This matches well with existing research where torula yeast has successfully replaced up to 40% of protein from fish meal in the diets of FW Atlantic salmon without negatively impacting growth performance^18^. The addition of torula yeast to a FM diet can even enhance the growth of FW Atlantic salmon compared to FM alone^13^. The present study provides further evidence that even in Atlantic salmon less than 2 g body weight, torula yeast is a suitable partial replacement for marine protein. The lack of difference in K across the different inclusion levels suggest torula yeast also provides comparable energy levels to marine protein^54^. Growth performance for fish fed MIX diets was best for fish fed the moderate inclusion MIX10, and K was comparable across all diets and fell within normal range for FW Atlantic salmon^53^. Few studies have explored the replacement of plant proteins with torula yeast despite the prevalence of these materials in commercial salmonid feed. The reduced growth performance of fish fed the MIX20 compared with MIX10 was also found when torula yeast replaced gluten and starch in a SBM diet also at an inclusion of 20%^13^, however MIX20 was comparable with the MIX00 control, suggesting MIX10 may be optimal for juvenile salmon. In comparison, when 25% inclusion level of torula yeast replaced a mixture of plant and marine protein sources in Atlantic Salmon through the freshwater to seawater transfer, growth performance and feed intake were improved compared to the control^14^. This difference in results could be due to the strain and growing conditions, suggesting this process can be optimised to improve the application for salmonids^18^. In an experiment with Tilapia (*Oreochromis mossambicus*) fed a mixed diet with combined animal, and plant protein, increasing levels of torula yeast showed a similar trend to this study, with moderate levels, in this case 30% torula yeast supporting better growth than lower or higher levels^55^. The present study suggests that there is potential for growth benefits with moderate inclusion levels of torula yeast as a replacement for plant protein. The decline in growth performance at higher inclusions levels could be driven by potential over-feeding of yeast presenting a detrimental impact^22^, however given that the higher inclusion is comparable with the control, a detrimental impact is unlikely in this study. This is supported by good growth performance of the 20% inclusion for the FM in this study. However, this study along with Øvrum Hansen et al., 2019, indicates that higher inclusion levels of torula yeast are more applicable in combination with a marine protein diet than they are with a mixed source protein diet, but that the strain and growth conditions could be improved to optimise utilisation in mixed diets^18^.

The gut microbiome of fish fed FM and MIX diets are altered by the replacement of conventional proteins with torula yeast, but the impact trends differ for the different dietary bases. The lack of difference in alpha diversity measures between any of the FM diets suggests that all three diets provide a comparable substrate to support a community with similar defining characteristics. However the actual community composition established is different, which is shown by the beta diversity, and this is most apparent at the genus level. The MIX diets support gut microbiome communities with different alpha diversity characteristics, especially for the highest inclusion level MIX20, and with different community compositions between the three inclusion levels which are elucidated at the genus level. Compared to the existing literature on the Atlantic salmon gut microbiome during the FW stage, the alpha diversity measures for both the FM and MIX fish in this study fall within normal levels for captive individuals^56^. The alpha diversity results of FM diets concur with comparable experiments with other salmonid species. In Rainbow trout (*O. mykiss*) kept in FW, up to 20% replacement of FM with the yeasts, *Saccharomyces cerevisiae* and *Wickerhamomyces anomalus,* did not significantly alter the gut microbial community diversity, but higher replacement levels of 40% and 60% reduced bacterial diversity, and even led to increasing presence of the pathogenic *Candida albicans* at 60% inclusion levels, and reduced the presence of LABs^40^. This suggests that inclusion of greater than 20% of yeasts may be problematic for salmonid health which could in turn affect production even when in combination with marine protein only^22^. To the knowledge of these authors this is the first experiment to assess the gut microbiome of Atlantic salmon when plant proteins in a mixed protein source diet are replaced with a yeast protein source, yet this is a highly relevant concern for commercial salmonid aquaculture. The impact to the alpha diversity was more pronounced than in marine protein diets for observed richness of ASVs, Shannon diversity and Chao1 diversity, which were all higher in the 0% control and moderate 10% inclusion of torula yeast than in the higher inclusion of 20% in the MIX diets. Higher levels of alpha diversity alone do not necessarily mean a healthier or more resilient community assemblage, and it will be important to confirm resilience through further assessment of health indicators^57^. In early stage Rainbow trout (*O.s mykiss),* alpha diversity measures decreased when animal protein was replaced with a mix of plant proteins at 50% and 97% replacement, much higher than in the present study^35^. This indicates changes in diet can be detected in the alpha diversity measures, suggesting that replacing plant proteins with torula yeast in our MIX diets influenced the community, more than replacing FM in a marine protein diet for our FM diets. In another monogastric animal, weanling pigs, a 40% replacement of conventional proteins (a combination of plant and marine proteins) with torula yeast (*C. jadinii*), both the alpha diversity and the beta diversity were significantly altered by the replacement, with lower alpha diversities at 40% replacment^41^, suggesting a similar trend as found in the present study for the MIX diets.

Existing research on the gut microbiome of FW Atlantic salmon suggests that even when alpha diversity is not altered, differences may be present in the beta diversity. Chao1 diversity and Shannon diversity were not altered by acute cold stress or a chronic environmental stress but the beta diversity was altered^58^. In early stage salmonids when the dietary protein composition is altered, existing studies observed a significant shift in the beta diversity and composition of the gut microbiome, in Atlantic salmon^59^, in Rainbow trout (*O. mykiss*)^35,60^, and in Arctic charr (*Salvelinus alpinus*)^61^. Similarly, in the present study, beta diversity was distinct between each inclusion level of torula yeast irrespective of the dietary base (FM or MIX). Since gut microbiota changes between freshwater and seawater transfer^31^, future studies should follow Atlantic salmon fed torula yeast as a dietary protein from first feeding to harvest to evaluate the impact across different life stages. In other cultured fish species, the presence of yeast species either as a supplement or as a protein replacement for conventional proteins also significantly altered the community compositions, in early-stage zebrafish (*Danio rerio*)^33^, in the gilthead sea bream (*Sparus aurata*)^62^ and the grass carp (*Ctenopharyngodon idellus*)^63^.

The gut microbiome composition of early-stage Atlantic salmon in this study for both FM and MIX diet bases, was similar at the phylum level regardless of the torula yeast inclusion level. Similarly in other studies that have used next generation sequencing in salmonids during the FW growth stages, the dominance of Firmicutes followed by Proteobacteria have been noted with varying levels of Actinobacteria depending on the study^31,40,64,65^. This indicates that a core phyla composition can be expected regardless of diet and influenced by a range of other factors^31^. The drivers of differing community compositions can be seen in this study at the genus level. The dominance of Firmicutes at the phyla level for both FM and MIX diets at all inclusion levels is explained by the high relative abundance of *Staphylococcus* at the genus level. This dominance of *Staphyloccocus* is consistent with other Atlantic salmon, and many other fish gut microbiome characterisations^66,67^, and it has been associated with nutritional processes in the salmonid, Arctic charr (*S. alpinus*)^68^. This study revealed different trends for FM and MIX diets with increasing inclusion of torula yeast, further evidence that torula yeast interacts differently with marine protein diets and mixed source protein diets. This difference could be due to differing levels of dietary fibre in FM and the MIX diets in this study. Torula yeast has a high total dietary fibre (TDF), at 20% TDF, higher than FM which is largely devoid of TDF but may see values as high as 5%^69^, and may explain why we see a slight trend toward increased diversity indices with increasing torula yeast inclusion, although the effect is minimal. Conversely in the MIX diets, a similarly high TDF from the combined plant meals^69^ is being replaced by the torula yeast. This suggests that TDF is not the only driver of the decreased diversity indices seen for the high yeast MIX20 diet. The torula yeast was 12% insoluble fibre and 8% soluble fibre (20% TDF) (R. Ekmay 2021, personal communication 26 May). Future investigations should assess the balance of soluble and insoluble fibre components of formulated feeds with torula yeast, and their impact on fish gut microbiota since dietary fibre is known to influence microbiome in other animals^70^. It will be important to elucidate the significance of such differing trends for health and development of cultured salmonids. The LABs from the genus *Weissella* and *Leuconostoc*^37^ slightly increased with increasing inclusion of torula yeast in FM diets. Conversely, for the MIX diets, the LABs *Lactobacillus*, *Leuconostoc*, and *Weissella*^37^ were present, and for the former two they declined with increasing inclusion of torula yeast, whereas the latter increased slightly in the presence of torula yeast. In the finfish literature, LABs are widely regarded to be linked to positive benefits such as disease resistance, improved performance and are associated with innate immune activities^37,71^, however the exact impact is dependent on the species of bacteria and the specific host. In the FM diets of this study, *Clostridium_sensu_stricto_1* and *Preptostreptococcus* were both higher in diets containing torula yeast, and highest in the modest 10% inclusion. These genera (*Clostridium* and *Preptostreptococcus*) have been associated with faster growth in Rainbow trout (*O. mykiss*) faecal bacteria samples, although in our study *Clostridium_sensu_stricto_7* did not show a strong trend. However, the genus *Paeniclostridium* was associated with slower growing individuals^72^ and this was also highest in FM10 of the fish from this study, presenting conflicting findings. Since growth performance was not significantly different in the FM diets of the present study, it might suggest that Atlantic salmon have different bacterial indicators of growth than other salmonids. In the MIX diets, the genus *Corynebacterium* was lowest in the moderate 10% inclusion of torula yeast, and highest in the 20% inclusion level, and in a Rainbow trout (*O.mykiss*) study, this genus was associated with slow growing individuals^72^, which does correspond with the growth results for the present study of Atlantic salmon, suggesting it may be a potentially useful indicator for both salmonid species. It would be highly valuable for future research to identify bacteria in the digestive tract of Atlantic salmon that are associated with fast and slow growing individuals.

This study indicates that torula yeast can effectively partially replace FM in a marine protein-based diet up to an inclusion of 20% without adverse impact to the gut microbiome community assemblage, and some potential benefits in terms of raising the levels of some LABs. Conversely, when torula yeast is added to a mix of protein sources it is more effective for growth performance and promoting desirable gut bacteria at moderate levels of 10% inclusion than at the higher inclusion level of 20%. This study sampled the gut digesta and mucosa together, which may have masked further differences in the gut microbiome community composition, since the digesta appears most impacted by diet. Future studies should focus on the digesta where possible^32^, although this has proved challenging with very small Atlantic salmon, it would also be valuable to investigate the gut microbiome down to the species taxonomic resolution. Additionally, this early developmental stage in the salmonid life cycle is a time of a highly dynamic gut microbiome that is malleable and has the potential to change when the dietary input changes and the impact of torula yeast may be different at later developmental stages, and wil be important to investigate^30,35^.

## Conclusions

Torula yeast (*Cyberlindnera jadinii*) has been identified as a promising alternative protein for Atlantic salmon feeds. This study has demonstrated that during the freshwater growth stage, torula yeast can partially replace conventional proteins in formulated feeds, but that the optimal level of inclusion may be dependent on the total dietary composition and the types of proteins that are being replaced. In a marine protein diet, this study revealed that 20% torula yeast can be included as a replacement for fish meal without altering growth performance and with possible benefits for gut microbial community such as an increase in some lactic acid bacteria. Comparatively, in a diet that combines marine protein and plant proteins, a 10% inclusion of torula yeast supported better growth performance than conventional proteins. At higher inclusion levels of 20%, there were no growth benefits and potentially adverse changes to the gut microbiome, such as a decrease in lactic acid bacteria and increasing levels of bacteria associated with slower growth in other salmonid species. Future research should investigate why torula yeast inclusion produces different results in combination with different dietary proteins, and the potential links between changes in the gut microbiome with growth and resilience in Atlantic salmon.

## Data Availability

The datasets generated for gut microbiome during the current study are available in PRJNA732903 repository, http://www.ncbi.nlm.nih.gov/bioproject/732903. Additional datasets used during the current study are available from the corresponding author on reasonable request.
